# A Green Lipophilization Reaction of a Natural Antioxidant

**DOI:** 10.3390/antiox12020218

**Published:** 2023-01-18

**Authors:** Valeria Pappalardo, Nicoletta Ravasio, Ermelinda Falletta, Maria Cristina De Rosa, Federica Zaccheria

**Affiliations:** 1 National Research Council-Institute of Chemical Sciences and Technology (CNR-SCITEC) “G. Natta”, Via Golgi 19, 20133 Milano, Italy; 2 Department of Chemistry, University of Milan, Via C. Golgi 19, 20133 Milano, Italy; 3 National Research Council-Institute of Chemical Sciences and Technology (CNR-SCITEC) “G. Natta”, Largo F. Vito, 1, 00168 Rome, Italy

**Keywords:** natural antioxidants, lipophilization, food waste valorization, sustainable chemistry

## Abstract

A natural antioxidant, widely spread in plants, chlorogenic acid (CGA), can be lipophilized through a heterogeneous, non-enzymatic, catalytic process. Thus, sulfonic resins under no solvent conditions allow to obtain a series of esters in up to 93% yield through reaction of CGA with fatty alcohols of different chain length. The reaction takes place in one single step under mild conditions with conversions up to 96% and selectivity up to 99%. Product recovery in high purity was very easy and the esters obtained were fully characterized with spectroscopic techniques and through the DPPH test to verify the preservation of antioxidant activity. According to this test, all of them showed increased activity with respect to the parent acid and anyway higher than butylated hydroxyanisole. An in-silico method also suggested their very low toxicity. The increased lipophilicity of the esters allows their formulation in cosmetic and nutraceutic lipid-based products.

## 1. Introduction

Antioxidants are a very important class of chemicals with a global market valued more than 3 billion $ in 2020 and supposed to grow significantly. Their main application is in the plastic, rubber, and latex industry followed by the fuel and lubricants, food and feed, pharma and personal care sectors. However, there are growing concerns on various synthetic antioxidants. In particular, butylated hydroxyanisole, BHA, an antioxidant preservative (E320) used since the 1940s not only as a food preservative, but also in food packaging, cosmetics, and even medicine and regarded as a ‘‘generally recognized as safe (GRAS)” compound, has been accused of being carcinogenic and could also be an endocrine disruptor as it was shown to induce apoptotic cell death in mouse testicular cells [1].

For this reason, there is a lot of interest in exploring potential application of molecules of natural origin as antioxidants. Rosemary extracts are already widely used to improve the oxidative stability of edible oils and prevent degradation of polyunsaturated fatty acids, but also sesame seed extract and green tea extract have proven to be effective [2]. On the other hand, bio-oils produced by pyrolysis of spruce woodchips were found to be competitive with butylated hydroxytoluene (BHT), another widely used synthetic antioxidant, in stabilizing biodiesel, in particular methyl linoleate from autoxidation, [3] while lignin was found to be very promising for the development of nature inspired sunscreen cosmetic lotion [4].

In the last years, due to the increasing relevance of circular economy driven practices, there has been a growing interest in extracting polyphenols also from side streams of the food industry. This is the case, e.g., of resveratrol from grape pomace [5,6].

**Figure 1:**
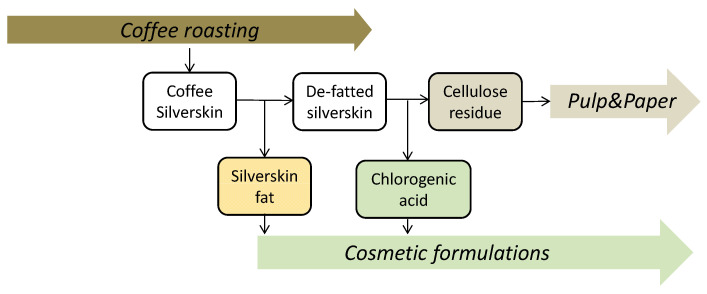
Scheme of silverskin upcycling within the CirCO Project.

In the framework of a recent project on the upcycling of silverskin [7], a side stream of the coffee roasting industry, we designed a cascade process involving the extraction of a fat with interesting cosmetic properties [8] followed by the extraction and functionalization of a powerful antioxidant, chlorogenic acid, and the use of the residual cellulose for the production of graphic paper (Figure 1) [9].

**Figure 2:**
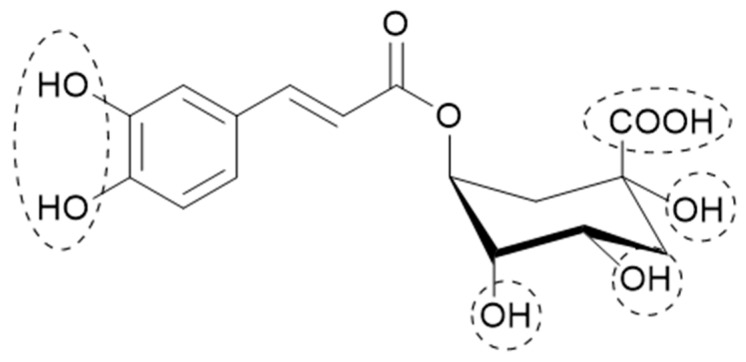
Chlorogenic acid structure and its main functionalities.

Chlorogenic acid (CGA), a mixture of isomers including 5-caffeoylquinic acid (5-CQA) as the most abundant, is a natural polyphenol with several pharmacological and biological activities including anti-aging properties [10]. This compound was found to have no cytotoxicity to fibroblasts and keratinocytes [11,12] and also detox activity towards lead-poisoned human fibroblast [13]. However, despite its several properties, the physiological activity of CGA in humans and its formulation in lipid-based products are precluded by a high hydrophilic structure. Thus, the presence of one carboxylic and five alcoholic groups (Figure 2) increases its hydrophilic character and hinders its use in pharmaceutical and nutraceutical preparations as well as in cosmetic products due to both poor miscibility with lipid-based formulations and low permeability through the skin tissues.

Lipophilization, the grafting of a lipophilic moiety onto a hydrophilic one, has long been recognized as a valuable tool to improve compatibility of CGA with lipid-based formulations [14,15,16]. Guyot et al. obtained a 60% yield with *n*-octanol after 30 days at 60 °C in the presence of a lipase [17]. On the other hand, López Giraldo et al. proposed an efficient, two-step method by first converting CGA into its methylester by means of an acidic resin and then transesterifying the latter with fatty alcohols of different chain lengths using a lipase-catalyzed reaction in a solvent-free medium. By this method, a yield up to 90% could be obtained in 96 h [15]. They also could show that esterification improves the antioxidant activity of 5-CGA [18]. Esterification of the 3- and 4-hydroxyl group of the quinic moiety was also obtained through reaction with palmitic acid in the presence of Novozyme 435 [16].

Structuring lipophilic phenolics in this way results in amphiphilic molecules with numerous combined beneficial properties and has been widely recognized to improve their oxidative stability and miscibility [19,20,21,22,23,24,25]. However, as already mentioned, up to now, only biocatalytic routes have been tested to convert CGA into its esters.

The chemical esterification of CGA is not trivial: it may oxidize in heated, or alkaline medium and several unwanted side reactions can take place reducing yield and requiring several purification steps.

Another way to lipophilize antioxidant is to make diacylglycerol esters. This has been realized in the case of rosmarinic [26] and ferulic acid [27] with a three-step process involving esterification of the acid with 1,2-O-isopropylidene glycerol, hydrolysis of the isopropylidene moiety, and lipase catalyzed transesterification with methyl or vinyl alkanoates of increasing chain length. This synthesis involves the use of triphenylphosphine and diisopropylazodicarboxylate in the first step (Mitsunobo reaction) toxic solvents and very long reaction times. Therefore, more sustainable processes are needed to bring the transformation on an industrial scale.

**Scheme 1:**
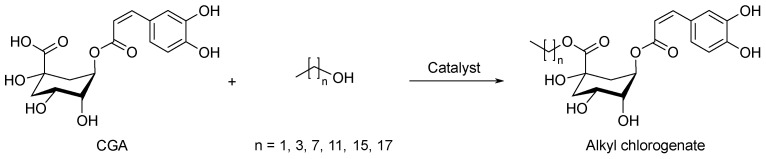
Lipophilisation of chlorogenic acid with different alcohols.

Here, we wish to report on the chemical lipophilization of CGA through a sustainable direct esterification with a fatty alcohol, in the presence of a heterogeneous catalyst and in the absence of solvent (Scheme 1).

## 2. Materials and Methods

### 2.1. Materials

All the reagents, solvents, and catalysts were purchased from Sigma-Aldrich (Merck Life Science S.r.l., Milano, Italy) and used without further purification.

### 2.2. General Synthesis of Acyl Chlorogenates

In a Schlenk tube CGA (1.063 g, 3 mmol, for the scale up), the catalyst (40% *w*/*w* with respect to CGA) and the proper amount of alcohol were added under argon atmosphere and a short magnetic stirring. The reactor was therefore immersed in an oil bath pre-warmed at 75 °C (in the presence of ethyl alcohol) or 80 °C. The mixture was stirred at 750 rpm for 6, 6, 16, or 40 h, respectively, for ethyl, butyl-, octyl-, and dodecyl alcohol, and it was finally analysed by TLC and HPLC.

A typical isolation procedure was the one described as follows for butyl-chlorogenate.

Butyl-chlorogenate. After 6 h, the reaction mixture was cooled to room temperature, diluted in warm ethyl acetate and filtered under vacuum with a Büchner funnel to remove the catalyst and the unreacted chlorogenic acid. After concentration in a rotary evaporator at 40 °C, the residual brown foamy solid was recrystallized from ethyl acetate (30 mL) to give a white-ivory powder, 97% pure, in 88% yield. TLC (*n*-hexane/ethyl ether/methanol, 4.5/4.0/1.5), Rf 0.41. The product was characterised by LC-MS and NMR.

Procedures for ethy-, octyl-, and dodecyl-chlorogenates are described in the Supplementary Information section.

### 2.3. HPLC Analysis

The HPLC analyses were performed by using an Agilent Technologies 1260 Infinity, (Santa Clara, CA, USA) equipped with a UV variable wavelength detector, and an Atlantis T3 Column (4.6 × 100 mm, 3 µm, 100 Å) column under the following conditions. Flow rate 1.0 mL/min, column temperature 40 °C, detection λ 313 nm. Before analysis, each sample (5–15 μL) was diluted in MeOH (2.0 mL) and injected (20 μL) with a 50 μL glass syringe (Agilent). The mobile phase consisted of a mixture of 0.1% formic acid in water (solvent A) and 0.1% formic acid in acetonitrile (solvent B). The elution program was a linear gradient from 2% to 95% B in 25 min, then isocratic at 5% A and 95% B for 10 min and finally a linear gradient from 95% to 2% B in 5 min to re-equilibrate the system. Conversions, yields, and selectivity were obtained by using the linear calibration curves (r^2^ > 0.99) of GCA and purified CGA-esters in concentration ranging from 1 × 10^−3^ to 1 × 10^−5^ mmol/mL).

A comparison of the HPLC analyses of the final crude reaction mixtures of the synthesized alkyl chlorogenates is reported in Figure S1.

### 2.4. LC-MS Analysis

The products identification was carried out by HPLC/MS analyses on a Thermo Fisher (Waltham, MA, USA) LCQ Fleet ion trap mass spectrometer equipped with a HPLC UltiMate™ 3000 system containing UV detector.

A Zorbax RX-C18 (2.1 × 150 mm-5 micron) column (Agilent, Santa Clara, CA, USA) was employed for the chromatographic separation. The products were separated using a gradient elution program, which consisted of water with 0.1% formic acid (Solvent A) and acetonitrile with 0.1% formic acid (Solvent B) at a steady flow rate of 0.25 mL/min. The column was maintained at the temperature of 35 °C. The injection volume was 2 µL for all the samples. The gradient started with 98% A and 2% of B for 10 min, then in 25 min the percentage of B ramped to 95% B, whereas A dropped to 5% and these values were maintained for 10 min. The compounds were monitored at 190 nm by the UV detector. The ion trap mass spectrometer operated both in negative and positive ion mode in full-scan mode in the mass/charge (m/z) range of 50–2000. MS interface conditions for sample acquisition in positive mode were the following: capillary temperature 275 °C, sheath gas flow rate (arb) 20, auxiliary gas flow rate (arb) 5, spray voltage 5 kV, capillary voltage 17 kV, and tube lens 75 V.

MS interface conditions for sample acquisition in negative mode were the following: capillary temperature 275 °C, sheath gas flow rate (arb) 20, auxiliary gas flow rate (arb) 10, spray voltage 4.50 kV, capillary voltage −5 V, tube lens –10 V.

### 2.5. NMR Analysis

High-resolution ^1^H and ^13^C NMR spectra were acquired at 400.13 and 100.62 MHz, respectively, on a Bruker Avance II 400 spectrometer (Bruker, Karlsruhe, Germany) interfaced with a workstation running a Windows operating system and equipped with a TOPSPIN 3.6 software package. Hence, 10 mg of ester was dissolved in 0.6 mL of MeOD and the spectra were recorded at 27 °C. Chemical shifts (δ) were given in parts per million (ppm) and referenced to the solvent signals (δH 2.50 and δC 39.50 ppm from Tetramethylsilane (TMS)). The ^13^C NMR signal multiplicities were based on attached proton test (APT) spectra and assigned on the basis of ^1^H-^13^C correlation experiments (Heteronuclear Multiple Quantum Correlation spectroscopy, HMQC, and Heteronuclear Multiple Bond Correlation spectroscopy, HMBC). The 1H signals were assigned by using ^1^H–^1^H correlation experiments (correlation spectroscopy, COSY, and total correlation spectroscopy, TOCSY). The following abbreviations are used in reporting NMR data: s = singlet; d = doublet; t = triplet; quint = quintet; dd = doublet of doublets; m = multiplet; br.s. = broad signal. The ^1^H NMR spectrum of ethylchlorogenate is reported in Figure S2 while the ^13^C APT NMR spectrum opf butylchlorogenate is reported in Figure S3.

### 2.6. ESI-MS Analysis

ESI-MS analyses was performed using a Shimadzu (Columbia, MD, USA) High Performance Liquid Chromatograph Mass Spectrometer (LCMS-2020 ESI/DUIS) equipped with a Bruker Esquire 3000 Ion Trap Plus. Each sample (1 mg) was dissolved in CH_3_CN (2 mL) and infused (10 µL) in the ESI source by using a 25 µL glass syringe. The mass spectrometer operating parameters were optimized as follows: interface voltage, 4.5 kV; nebulizer gas flow, 3 L/min; drying gas flow, 15 L/min; desolvation line (DL) temperature, 250 °C; heat block temperature, 400 °C in ESI source. The interface temperature was 350 °C.

ESI-MS of ethyl chlorogenate, mw 382.37 g/mol. Positive-ion mode, m/z: 446.15 [M + CH_3_CN + Na^+^], 487.20 [M + 2CH_3_CN + Na^+^], 787.35 [2M + Na^+^]. Negative ion-mode, m/z: 381.15 [M − H_+_], 763.45 [2M − H_+_].

ESI-MS spectra of *n*-butyl chlorogenate, mw 410.42 g/mol. Positive-ion mode, m/z: 411.20 [M + H^+^], 474.20 [M + CH_3_CN + Na^+^], 515.25 [M + 2CH_3_CN + Na^+^], 823.45 [2M + Na^+^]. Negative ion-mode, m/z: 409.20 [M − H^+^], 819.45 [2M − H^+^].

ESI-MS spectra of *n*-octyl chlorogenate, mw 466.22 g/mol. ESI-MS. Positive-ion mode, m/z: 467.25 [M + H^+^], 530.30 [M + CH_3_CN + Na^+^], 571.35 [M + 2CH_3_CN + Na^+^], 955.60 [2M + Na^+^]. Negative ion-mode, m/z: 465.25 [M − H^+^], 931.60 [2M − H^+^] (Figure S4).

ESI-MS spectra of *n*-dodecyl chlorogenate, mw 522.64 g/mol. ESI-MS. Positive-ion mode, m/z: 523.35 [M + H^+^], 586.40 [M + CH_3_CN + Na^+^], 1067.75 [2M + Na^+^]. Negative ion-mode, m/z: 521.40 [M − H^+^], 1043.75 [2M − H^+^] (Figure S5).

### 2.7. 1,1-Diphenyl-2-picrylhydrazyl (DPPH) Radical Scavenging Activity

The DPPH radical scavenging ability was evaluated following the slightly modified methods of López-Giraldo et al. [15] and Ahmad et al. [28] Briefly, 2.0 mL of an ethanol solution of DPPH (60 µM) was placed in 1 cm quartz cuvette and the absorbance was read at 517 nm against pure ethanol. After 3 s, 50 µL of an ethanol solution of sample, S, (0.154–1.15 mM) was added and quickly mixed. Finally, the decay of absorbance was monitored each 2 s for 1 h at room temperature by using a Shimadzu’s UV-3600 Plus spectrophotometer (Shimadzu Corp., Kyoto, Japan) equipped with software Spectramenager (Fixed Wavelength Measurement). The S concentrations were chosen to have a S/DPPH molar ratio ranging from 0.10 to 0.5. Trolox, ascorbic acid and BHA were used as positive controls. The addition of pure ethanol (50 μL) instead of S was used as blank to avoid a scavenging activity overestimation due to intrinsic decreasing of DPPH absorbance. All assays were performed in triplicate. The right sample absorbance at time *t*, *Abs(t)*, was calculated using the Equation (1):

*Abs(t)* = *Abs(s)* + *(Abs(b)t*_0_ − *Abs(b)t)* (1)

where *Abs(s)* is the measured absorbance at time *t*, while *Abs(b)t*_0_ and *Abs(b)t* are the absorbance of blank at time zero and time *t*, respectively S1.

The residual DPPH percentage, % DPPH(t), for each time was calculated by the Equation (2):

Residual% DPPH(t) = [DPPH(s)/DPPH(t_0_)] * 100 (2)

where DPPH(s) and DPPH(t_0_) are the concentration of DPPH at time *t* and time zero, respectively, estimated through a calibration curve, [DPPH] = (*Abs* − 0.0643)/(0.0089) R2 = 0.997, obtained by measuring the absorbance of 5 ethanol solutions of DPPH (10–80 µmol·L^−1^) at 517 nm S2.

The antioxidant activity was assessed as the amount of sample needful to decrease the initial DPPH concentration by 50% (EC50, µmol of sample/µmol of DPPH) and it was calculated by plotting the percentage of the residual unoxidized DPPH against the S/DPPH molar ratio. The antioxidant activity was also expressed as Trolox equivalent antioxidant capacity (TEAC, µmol of Trolox/µmol of sample/µmol of DPPH in the reaction medium) by using the Equation (3).

TEAC = EC50(Trolox − µmol/L)/EC50(sample − µmol/L)(3)

### 2.8. Toxicity Prediction

The Toxicity Prediction by Komputer-Assisted Technology (TOPKAT) module of Discovery Studio 4.0 (BIOVIA, Dassault Systèmes (2018) Discovery Studio, Dassault Systèmes, San Diego, CA, USA) was used to assess the toxicity of synthesized compounds. Ames mutagenicity, NTP carcinogenicity, and skin irritancy were predicted to evaluate the probability that the candidates are non-carcinogen, non-mutagen, and non-toxic.

## 3. Results

### 3.1. Chlorogenic Acid Lipophilisation

As already mentioned in the chemical esterification of CGA high temperatures and alkaline conditions should be avoided as the substrate is prone to oxidation under these conditions and several unwanted side reactions can take place.

As far as acid catalyzed reactions are concerned, Fischer esterification with methanol and ethanol in the presence of sulfuric acid was reported to give low yields, 59% and 35% respectively [29]. This method requires time consuming work up and production of inorganic salts.

The use of solid acid catalysts in esterification reactions was already reported [30,31,32]. However, both inorganic mixed oxides and polyoxometallates require temperatures in the range 160–200 °C and therefore appear unsuitable for the esterification of CGA. Therefore, we examined a series of sulfonic resins at 80 or 100 °C in the reaction of CGA with n-octanol. The use of organic resins has actually been explored for esterification reactions in other kind of applications, such as in biomass derived platform molecules functionalisation [33,34]. In order to avoid the use of a solvent, the reacting alcohol was used in the minimal amount required to stir the reaction mixture. Results are reported in Table 1.

All the tested resins but Aquivion PW98S gave excellent results in terms of conversion. Also, selectivity to the desired product was high at least at 80 °C. The low conversion observed with PW98 may be due to low acid sites density while in the case of Amberlite 120 and AqP65 the lower density is balanced by the very high strength of acidic groups.

The very high selectivity observed with Amberlyst 15 allowed to reach a very high yield in the desired product and therefore this resin under these conditions was used for the esterification of other alcohols. Results are reported in Table 2. Excellent results were obtained for the esterification with ethanol and butanol. However, as long as the fatty alcohol carbon atom chain length increases, the reaction time also increases very much while selectivity decreases. No water removal during reaction was needed and the product was separated in a very simple way (ESI).

The method here proposed allows to reach yields even higher than 90% in one single step under no solvent conditions and in short reaction times.

The antioxidant activity of the esters obtained in this way was tested through both the DPPH test and the Trolox one and compared to the activity of pure CGA.

### 3.2. DPPH Scavenging Radical Activity and Toxicity Predictive Evaluation

Many chronic and degenerative diseases arise from free radical reactive oxygen species (ROS) that are produced by oxidative stress. Phenolic compounds, ubiquitous chemicals in plants, are able to counteract oxidative damage by inhibiting the enzymes responsible for the production of ROS and therefore display high antioxidant ability and free radical scavenging capacity [35]. Several methods are routinely used to evaluate the efficiency of antioxidants. Among them, the DPPH assay is one of the best-known.

In the present study, the antiradical behavior of the synthesized esters against DPPH was evaluated at fixed reaction time (10 min). It was compared to that of pure CGA, ascorbic acid (Vit C) and butylated hydroxyanisole (BHA) both recognized as safe (GRAS) for use as antioxidant and chemical preservative in foods, cosmetics, and medicines.

**Figure 3:**
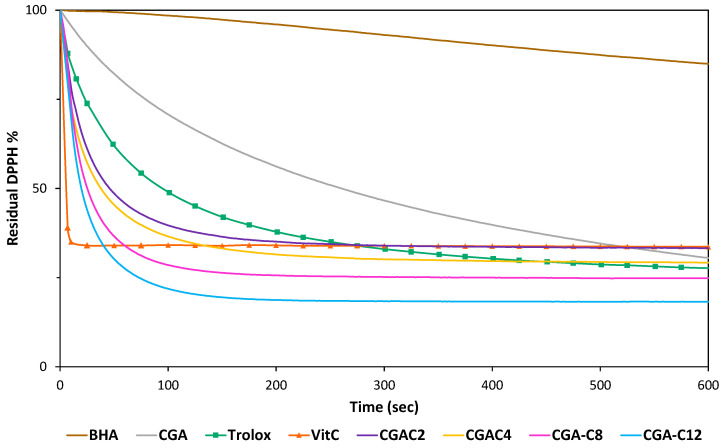
DPPH radical scavenging activity of synthesized alkyl chlorogenates, pure chlorogenic acid, and the commonly used antioxidants Trolox, ascorbic acid (VitC) and butylated hydroxyanisole (BHA). The curves express the percent remaining DPPH radical in the time, calculated as a function of the molar ratio of sample/DPPH, 0.30.

The first value considered was the “half maximal effective concentration” (EC_50_) which is the amount of sample needful to halve the initial DPPH radical concentration. The lowest EC_50_, the highest antioxidant activity. Since several parameters affect this value, the EC_50_ of the examined sample is usually compared to the EC_50_ of Trolox (a water-soluble analog of α-tocopherol, accepted as benchmark to compare various substances or mixture) and expressed as Trolox equivalent antioxidant capacity (TEAC) [36]. The higher TEAC value, the higher radical scavenging ability [37]. Thus, results reported in Table 3 and Figure 3 show that at a fixed reaction time (10 min) all the esters exhibit a higher antioxidant activity with respect to that of CGA itself, ascorbic acid, and BHA. Moreover, the scavenging activity of *n*-octyl and *n*-dodecyl chlorogenates, 0.173 and 0.163 respectively, is higher than that of Trolox. The stronger activity of *n*-octyl and *n*-dodecyl chlorogenates could be due to the higher lipophilization. In fact, the longer alkyl chain makes a more flexible structure with a weaker intermolecular hydrogen bond between the ion pairs, thanks to the steric hindrance effect. Consequently, the access to the radical center is easier, the hydrogen transfer atom faster, and the scavenging activity higher [28,38,39].

Therefore, results obtained with DPPH showed that all the esters exhibit a higher antioxidant activity with respect to that shown by CGA itself.

Finally, the toxicity of these new antioxidants was investigated. A preliminary, predictive evaluation of this parameter is of paramount relevance in view of the industrial exploitation of the results. Up to now, all regulatory methods to determine acute oral toxicity are based on animal tests where the acute lethal dose to 50% of the treated animals (LD50 value) is typically used to assess hazard and regulatory classification.

Alternatives to animal tests, such as in silico methods, are strongly sought after. Therefore, we predicted a few toxicity indicators using the Discovery Studio module TOPKAT (Table 4).

The program employs the cross-validated quantitative structure toxicity relationship (QSTR) models for evaluating toxicity and uses optimal prediction space (OPS) validation methods for the interpretation of results [40]. Probability values from 0.0 to 0.30 are likely to produce a negative response in an experimental assay; whereas probability values greater than 0.70 are likely to produce a positive response in an experimental assay. Probabilities greater than 0.30 but less than 0.70 are considered indeterminate. Mutagenicity and carcinogenicity have been estimated together with skin irritancy in view of a possible cosmetic application in anti-age formulations.

Probability values of 0.000 from the Ames mutagenicity model indicate that all the CGA esters are likely to produce a non-mutagenic effect. Studies on female mouse showed no sign of carcinogenicity with computed probability of 0.000 for all the compounds. The computed probability values of 0.985 and 1.000 for the male mouse NTP carcinogenicity of C8 and C12 is greater than 0.70 and suggests that further investigation is needed. The computed probability for the skin irritation model ranges from 0.000 to 0.012, implying non-irritation.

## 4. Conclusions

A simple and green procedure for the lipophilization of Chlorogenic acid, an antioxidant widespread in plants, particularly in coffee beans and coffee silverskin, has been set up. The direct esterification reactions of CGA with different fatty alcohols is carried out under no solvent conditions in the presence of a sulphonic resin as acid catalyst with very high conversions and high selectivity allowing one to reach yields up to 93% in the case of the ethyl and octyl-esters. The work up procedure is very simple, and no chromatographic separation is needed due to the high yield in the products. Most remarkably, the antioxidant activity increases with the length of the lipophilic chain of the ester as well as its miscibility with lipid-based matrixes. A predictive model allows to exclude significant toxicity effects, particularly as far as skin irritation and skin sensitivity are concerned, thus paving the way to the use of CGA esters in anti-age cosmetic formulations. This lipophilization process can thus boost the upcycling of agri-food waste, a valuable tool for the transition towards a circular economy.

## Figures and Tables

**Table 1:** Esterification of CGA with n-octanol in the presence of different acidic resins.

	T (°C)	Ion Exch. Cap. (meq H+/g)	Conv.%	Yield%	Sel.%
Dowex dried	80	4.8	97.8	71.6	73.2
Amberlyst 16	100	4.8	96.4	50.8	52.7
Amberlyst 16	80	4.8	96.4	78.5	81.4
Amberlyst 15	80	4.7	94.8	93.4	98.5
Amberlite IR120	100	1.8	97.0	85.5	88.1
Aquivion PS65 S	80	1.48	93.7	80.4	85.9
Aquivion PW98 S	80	0.98	45.4	43.5	96.1

**Table 2:** Esterification of CGA with different alcohols in the presence of Amberlyst 15.

Alcohol	T (°C)	Time (h)	Conv.%	Yield%	Sel.%
Etyl-	75	6	94.3	93.8	99.5
Butyl-	80	6	96.4	90.6 ^a^	94.0
*n*-Octyl-	80	16	94.8	93.4	98.5
Lauryl-	80	40	93.4	76.4	81.8
Cetyl-	80	48	91.5	66.0	72.2
Steryl-	80	60	96.9	62.9	64.9

^a^ = isolated yield.

**Table 3:** Antioxidant activity of CGA esters at fixed reaction time (10 min).

Compound	EC_50_^a^	TEAC ^b^
Chlorogenic acid	0.214	0.86
Ethyl chlorogenate	0.203	0.91
Butyl chlorogenate	0.201	0.92
Octyl chlorogenate	0.173	1.06
Dodecyl chlorogenate	0.163	1.13
Ascorbic acid	0.203	0.91
Butylhydroxyanisole	0.362	0.51
Trolox	0.184	1.00

^a^ Half maximal effective concentration, µmol_S_/µmol_DPPH_; ^b^ Trolox equivalent, mmolTrolox/mmolsample/mmolDPPH.

**Table 4:** Toxicity prediction of the synthesized compounds expressed as calculated probability.

Compound	Ames Mutagenicity	Male Mouse NTP ^a^	Female Mouse NTP ^a^	Skin Irritation
chlorogenic	non-mutagen (0.000)	non-carcinogen (0.005)	non-carcinogen (0.002)	non-irritant (0.000)
C2	non-mutagen (0.000)	non-carcinogen (0.113)	non-carcinogen (0.000)	non-irritant (0.004)
C4	non-mutagen (0.000)	indeterminate (0.431)	non-carcinogen (0.000)	non-irritant (0.012)
C8	non-mutagen (0.000)	carcinogen (0.985)	non-carcinogen (0.000)	non-irritant (0.002)
C12	non-mutagen (0.000)	carcinogen (1.00)	non-carcinogen (0.000)	non-irritant (0.000)

^a^ NTP: National Toxicology Program.

## Data Availability

Not applicable.

## Supplementary Materials

The following supporting information can be downloaded at: https://www.mdpi.com/article/10.3390/antiox12020218/s1, Details about Purification procedures for chlorogenates and NMR Spectra; Figure S1. Comparison of the HPLC analyses of the final crude reaction mixtures of the synthesized alkyl chlorogenates; Figure S2. ^1^H NMR spectra (400.13 MHz) of ethyl chlorogenate, acquired in CD_3_OD-d_6_ at 27 °C; Figure S3. ^13^C APT NMR spectra (100.62 MHz) of butyl chlorogenate, acquired in CD_3_OD-d_6_ at 27 °C; Figure S4. ESI-MS spectra of *n*-octyl-chlorogenate, mw 466.22 g/mol; Figure S5. ESI-MS spectra of *n*-dodecyl-chlorogenate, mw 522.64 g/mol.
