# Supplementary material for: A Green Lipophilization Reaction of a Natural Antioxidant
Source: Antioxidants (Basel). 2023 Jan 18;12(2):218. doi: 10.3390/antiox12020218 (PMC9952416; doi:10.3390/antiox12020218)
Supplement: Supplementary file 1 [file antioxidants-12-00218-s001.zip › antioxidants-2088903-supplementary.pdf]

Antioxidants

Supplementary Experimental Information for

A Green Lipophilization Reaction of a Natural Antioxidant

Valeria Pappalardo [1], Nicoletta Ravasio [1,*], Ermelinda Falletta [2], Maria Cristina De Rosa [3] and Federica Zaccheria [1]

[1] National Research Council-Institute of Chemical Sciences and Technology (CNR-SCITEC) "G. Natta", Via Golgi 19, 20133 Milano, Italy
[2] Department of Chemistry, University of Milan, Via C. Golgi 19, 20133 Milano, Italy
[3] National Research Council-Institute of Chemical Sciences and Technology (CNR-SCITEC) "G. Natta", Largo F. Vito, 1, 00168 Rome, Italy
* Correspondence: nicoletta.ravasio@scitec.cnr.it; Tel.: +39-02-50314382

Table of Contents

Purification procedures for chlorogenates S3

Details about DPPH radical scavenging activity S3

NMR Spectroscopy S4

ESI-MS analyses S5

HPLC chromatograms S5

Selected NMR and ESI-MS spectra S6

Supplementary Experimental Information

Purification procedures for chlorogenates

Ethyl-chlorogenate. After 6 h mixing at 75 °C, the reaction mixture was cooled to room temperature, diluted in warm ethyl acetate and filtered under vacuum with a Büchner funnel to remove the catalyst and the unreacted chlorogenic acid. After concentration in a rotary evaporator at 40 °C, the residual brown foamy solid was solubilized using ethyl acetate/diethyl ether 4/1 (24 ml) and decanted to remove an insolubilized black by-product. The supernatant was dried again under vacuum and the residual solid was crystallized from ethyl acetate/diethyl ether 5/1 (12 ml) for 24 h at 4 °C to give a tiny white-ivory powder, 98% pure, in 70% yield. TLC (*n*-hexane/ethyl ether/methanol, 4.5/4.0/1.5) Rf 0.18.
Octyl-chlorogenate. The crude of reaction was diluted in acetone, passed through a Büchner funnel, to remove the catalyst, and concentrated under vacuum using a rotary evaporator at 40 °C. Then, the residual yellow-brown oil was washed three times with acetonitrile (7 ml), decanted to remove the unreacted alcohol and, finally, recrystallized from acetonitrile (35 ml). Octyl-chlorogenate was isolated as an ivory crystalline solid, 97% pure, in 80% yield. TLC (*n*-hexane/ethyl ether/methanol, 4.5/4.0/1.5) Rf 0.38.
Dodecyl-chlorogenate. After cooling to room temperature, the reaction mixture was diluted in acetone, passed through a cotton wool in a glass funnel and concentrated in rotary evaporator at 40 °C. Then, the residual brown oil was washed with *n*-hexane and centrifuged several times up to the removal of dodecyl alcohol excess, verified by TLC (*n*-hexane/ethyl ether/methanol, 4.5/4.0/1.5) Rf 0.49. Successively, the solid was crystalized with ethyl ether/*n*-hexane (1/0.5). Briefly, the solid was solubilized by using warm ethyl ether (35 ml at 30 °C), then *n*-hexane (20 ml) was added stirring few seconds and left at 20 °C for 24 h. The precipitate was finally filtered under vacuum with a Büchner funnel and washed with *n*-hexane. Dodecyl-chlorogenate was isolated as a fine ivory powder, 95% pure, in 50% yield.

NMR Spectroscopy

High-resolution ^1H and ^{13}C NMR spectra were acquired at 400.13 and 100.62 MHz, respectively, on a Bruker Avance II 400 spectrometer (Bruker, Karlsruhe, Germany) interfaced with a workstation running a Windows operating system and equipped with a TOPSPIN software package. 10 mg of ester was dissolved in 0.6 ml of MeOD and the spectra were recorded at 27 °C. Chemical shifts (δ) were given in parts per million (ppm) and referenced to the solvent signals [δ_H 2.50 and δ_C 39.50 ppm from Tetramethylsilane (TMS)]. ^{13}C NMR signal multiplicities were based on attached proton test (APT) spectra and assigned on the basis of ^1H-^{13}C correlation experiments (Heteronuclear Multiple Quantum Correlation spectroscopy, HMQC, and Heteronuclear Multiple Bond Correlation spectroscopy, HMBC). ^1H signals were assigned by using ^1H–^1H correlation experiments (Correlation SpectroscopY, COSY, and TOtal Correlation SpectroscopY, TOCSY).
The following abbreviations are used in reporting NMR data: s = singlet; d = doublet; t = triplet; quint = quintet; dd = doublet of doublets; m = multiplet; br.s. = broad signal.

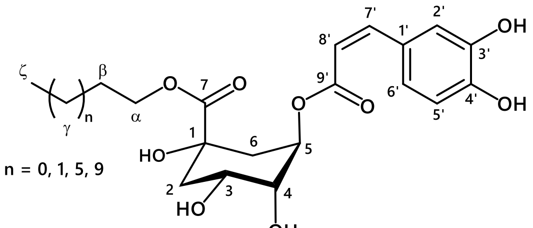

Ethyl chlorogenate, CGA-C2. ^1H NMR (400 MHz, CD_3OD) δ (ppm). 7.55 (d, 1H, J = 15.9 Hz, C=H-7'), 7.06 (d, 1H, J = 2.0 Hz, C=H-2'), 6.96 (dd, 1H, J = 8.2, 2.0 Hz, C=H-6'), 6.80 (d, 1H, J = 8.2 Hz, C=H-5'), 6.24 (d, 1H, J = 15.9 Hz, C=H-8'), 5.30 (td, 1H, J = 7.6, 4.6 Hz, CH-5), 4.22 – 4.10 (m, 3H, CH-3 + CH-α), 3.75 (dd, 1H, J = 7.5, 3.1 Hz, CH-4), 2.29 –2.03 (m, 4H, CH2-6 and CH2-2), 1.26 (t, 3H, J = 7.1 Hz, CH-β). **^{13}C NMR (101 MHz, CD_3OD) δ (ppm).** 173.58 (C=O 7), 166.93 (C=O 9'), 148.28 (C-3'), 145.78 (C-7'), 145.46 (=C-4'), 126.27 (C-1'), 121.58 (=C-6'), 115.15 (=C-5'), 113.73 (=C-2'), 113.67 (=C-8'), 74.37 (C-1), 71.25 (C-4), 70.77 (C-3), 69.00 (C-5), 61.15 (C-α), 36.60 (C-6), 36.38 (C-2), 12.90 (C-β).

Butyl chlorogenate, CGA-C4. **^{1}H NMR (400 MHz, CD_3OD) δ (ppm).** 7.54 (d, 1H, *J* = 15.9 Hz, C=H-7'), 7.06 (d, 1H, *J* = 2.0 Hz, C=H-2'), 6.97 (dd, 1H, *J* = 8.2, 2.0 Hz, C=H-6'), 6.80 (d, 1H, *J* = 8.2 Hz, C=H-5'), 6.23 (d, 1H, *J* = 15.9 Hz, C=H-8'), 5.29 (d, 1H, *J* = 5.2 Hz, CH-5), 4.19 – 4.04 (m, 3H, CH-3 + CH-α), 3.75 (dd, 1H, *J* = 7.3, 3.1 Hz, CH-4), 2.28 – 2.02 (m, 4H CH_2-6 and CH_2-2), 1.72 – 1.56 (m, 2H, CH-β), 1.45 – 1.25 (m, 2H, CH_2- γ), 0.91 (m, 3H, CH_3-ζ). **^{13}C NMR (101 MHz, CD_3OD) δ (ppm).** 173.66 (C=O 7), 166.87 (C=O 9'), 148.30 (C-3'), 145.82 (C-7'), 145.47 (=C-4'), 126.25 (C-1'), 121.57 (=C-6'), 115.14 (=C-5'), 113.74 (=C-2'), 113.65 (=C-8'), 74.34 (C-1), 71.07 (C-4), 70.80 (C-3), 68.82 (C-5), 64.98 (C-α), 36.63 (C-6), 36.24 (C-2), 30.22 (C-β), 18.68 (C-γ), 12.59 (C-ζ).

***n*-Octyl chlorogenate, CGA-C8. ^{1}H NMR (400 MHz, CD_3OD) δ (ppm).** 7.52 (d, 1H, *J* = 15.9 Hz, C=H-7'), 7.04 (d, 1H, *J* = 1.9 Hz, C=H-2'), 6.94 (dd, 1H, *J* = 8.2, 1.9 Hz, C=H-6'), 6.78 (d, 1H, *J* = 8.2 Hz, C=H-5'), 6.20 (d, 1H, *J* = 15.9 Hz, C=H-8'), 5.26 (d, 1H, *J* = 6.5 Hz, CH-5), 4.14 (dt, 1H, *J* = 7.1, 3.4 Hz, CH-3), 4.06 (td, 2H, td, *J* = 6.8, 3.3 Hz, CH-α), 3.74 (dd, 1H, *J* = 7.0, 3.1 Hz, CH-4), 2.29 – 1.98 (m, 4H CH_2-6 and CH_2-2), 1.67 – 1.54 (m, 2H CH-β), 1.31 – 1.15 (m, 10H, CH2 chain γ), 0.87 (t, 3H, *J* = 6.9 Hz, CH_3-ζ). **^{13}C NMR (101 MHz, CD_3OD) δ (ppm).** 173.63 (C=O 7), 166.78 (C=O 9'), 148.34 (C-3'), 145.85 (C-7'), 145.49 (=C-4'), 126.19 (C-1'), 121.57 (=C-6'), 115.11 (=C-5'), 113.71 (=C-2'), 113.57 (=C-8'), 74.19 (C-1), 70.84 (C-4), 68.60 (C-3), 68.59 (C5), 65.24 (C-α), 36.59 (C-6), 36.04 (C-2), 31.47, 28.87, 28.86 (3 CH_2 chain γ), 28.12 (C-β), 25.50 and 22.27 (2 CH_2 chain γ), 13.01 (C-ζ).

***n*-Dodecyl chlorogenate, CGA-C12. ^{1}H NMR (400 MHz, CD_3OD) δ (ppm).** 7.54 (d, 1H, *J* = 15.9 Hz, C=H-7'), 7.06 (t, 1H, *J* = 5.7 Hz, C=H-2'), 6.96 (dd, 1H, *J* = 8.2, 2.0 Hz, C=H-6'), 6.80 (d, 1H, *J* = 8.2 Hz, C=H-5'), 6.22 (d, 1H, *J* = 15.9 Hz, C=H-8'), 5.29 (dd, 1H, *J* = 12.8, 6.0 Hz, CH-5), 4.22 – 4.13 (m, 1H, CH-3), 4.13 – 4.03 (m, 2H, CH-α), 3.75 (dd, 1H, *J* = 7.1, 3.1 Hz, CH-4), 2.31 – 1.93 (m, 4H CH_2-6 and CH_2-2), 1.63 (dd, 2H, *J* = 14.4, 7.1 Hz, CH-β), 1.31 – 1.23 (m, 16H, CH_2 chain γ), 0.91 (dd, 3H, *J* = 8.3, 5.4 Hz, CH_3-ζ). **^{13}C NMR (101 MHz, CD_3OD) δ (ppm).** 173.64 (C=O 7), 166.81 (C=O 9'), 148.32 (C-3'), 145.84 (C-7'), 145.49 (=C-4'), 126.22 (C-1'), 121.56 (=C-6'), 115.11 (=C-5'), 113.74 (=C-2'), 113.61 (=C-8'), 74.25 (C-1), 70.93 (C-4), 70.83 (C-3), 68.68 (C-5), 65.24 (C-α), 36.61 (C-6), 36.16 (C-2), 31.65, 29.33, 29.31, 29.20, 29.05, 28.87 and 28.88 (7 CH_2, chain γ), 28.11 (C-β), 25.50 and 22.31 (2 **C**H_2 chain γ), 13.01 (C-ζ)

HPLC chromatograms

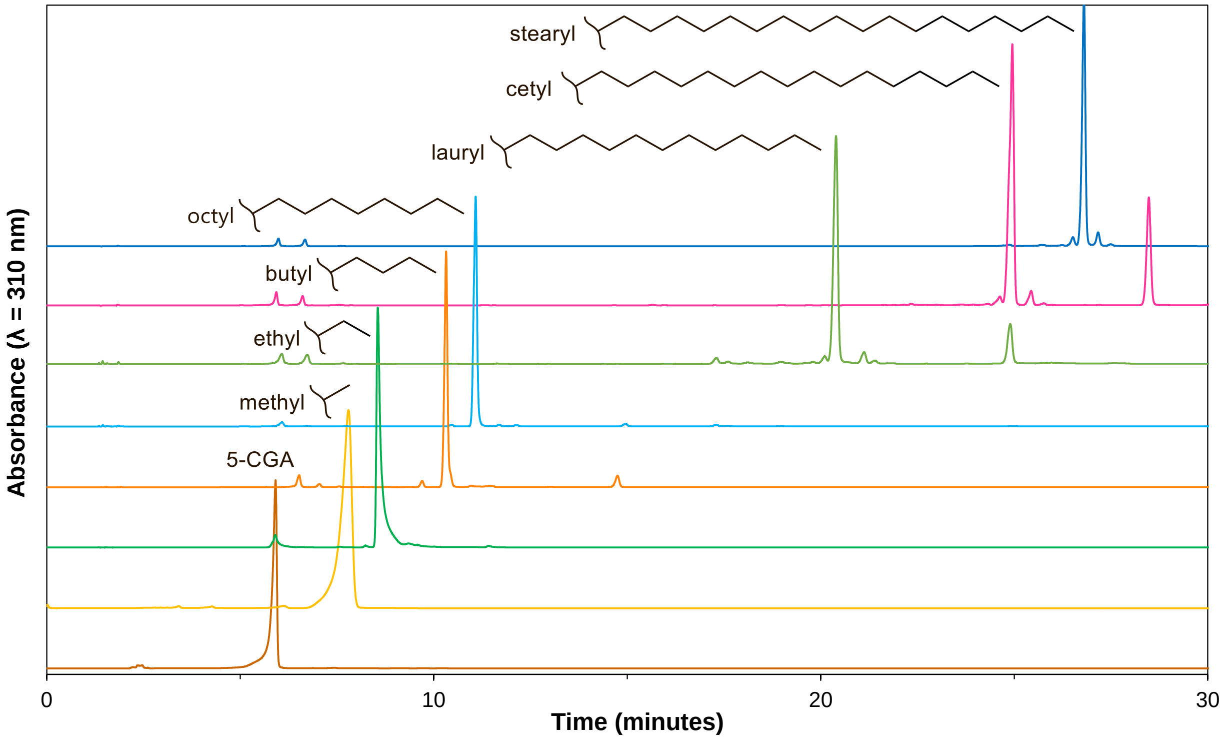

Figure S1. Comparison of the HPLC analyses of the final crude reaction mixtures of the synthesized alkyl chlorogenates. Mobile phase: 0.1% formic acid in water (Solvent A) and 0.1% formic acid in acetonitrile (Solvent B). Elution program: linear gradient from 2% to 95% B in 25 min, then isocratic at 5% A and 95% B for 10 min and finally linear gradient from 95% to 2% B in 5 min.

NMR Spectra

Ethyl chlorogenate, CGA-C2

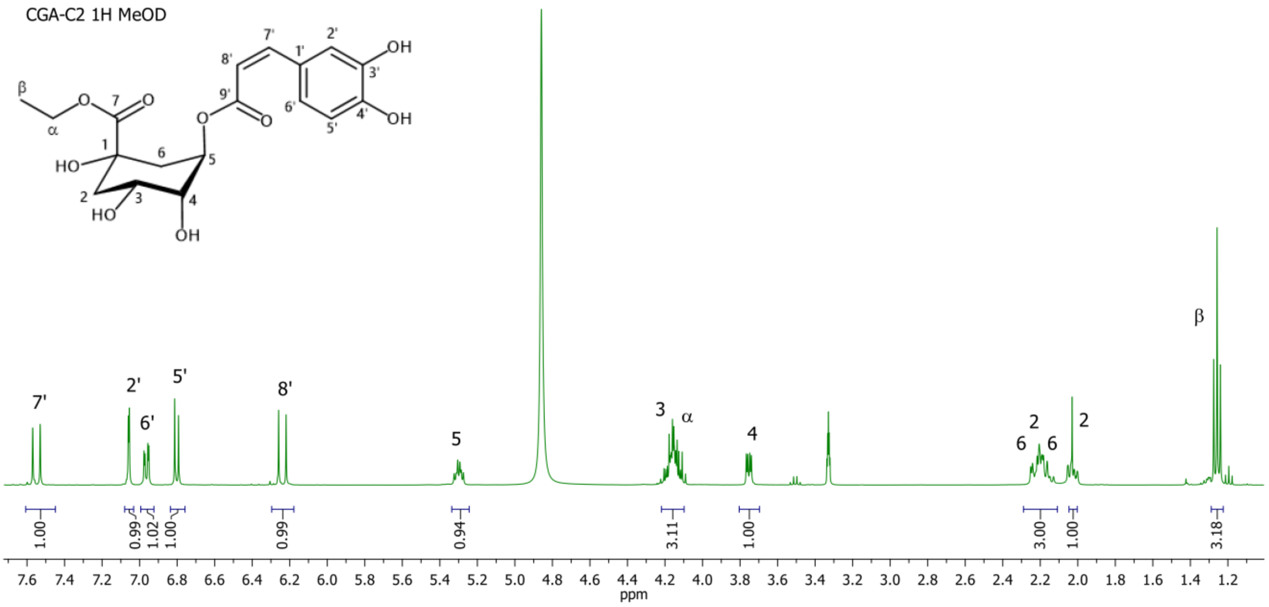

Figure S2. ^{1}H NMR spectra (400.13 MHz) of ethyl chlorogenate, acquired in CD_3OD-d_6 at 27 °C.

Butyl chlorogenate CGA-4

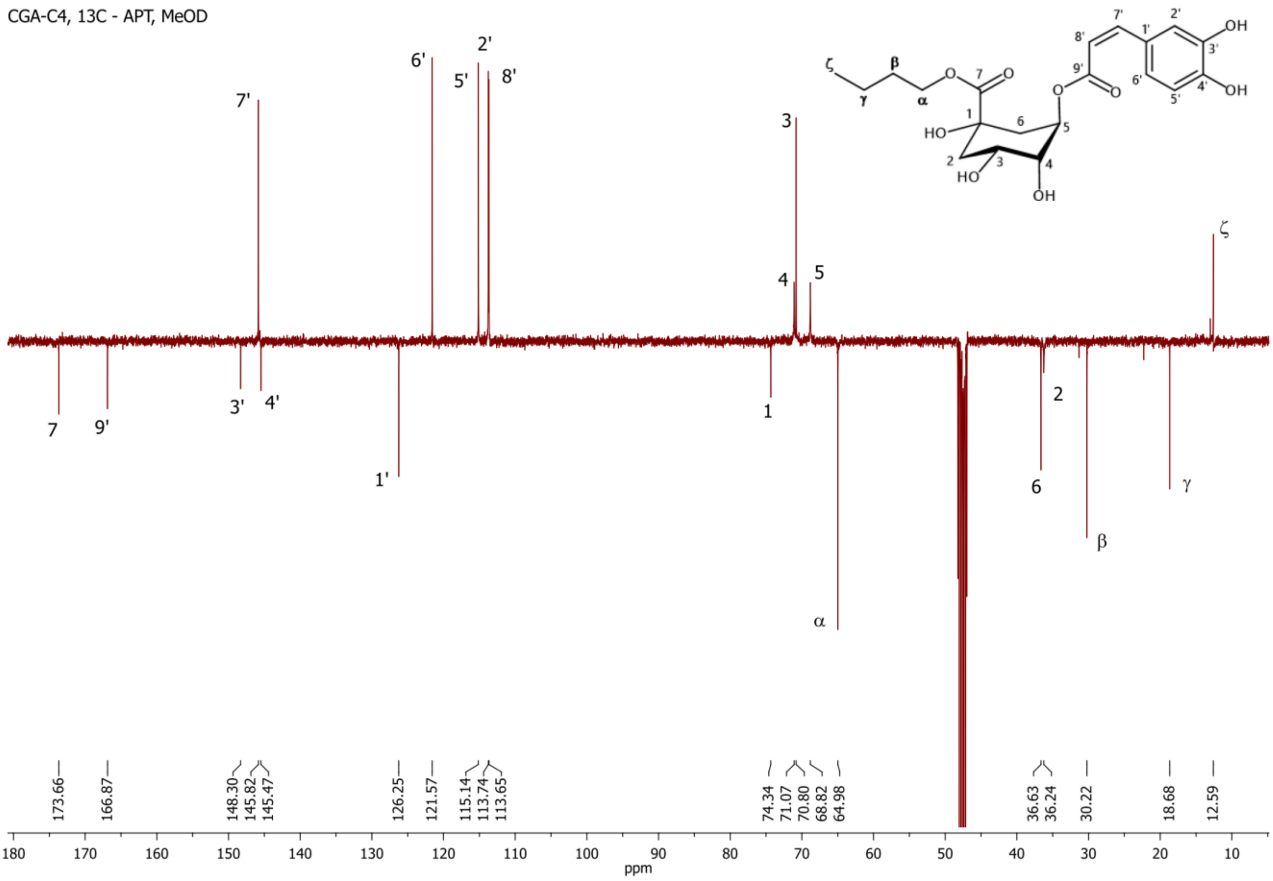

Figure S3. ^{13}C APT NMR spectra (100.62 MHz) of butyl chlorogenate, acquired in CD_3OD-d_6 at 27 °C.

ESI- Mass Spectra

n-octyl chlorogenate

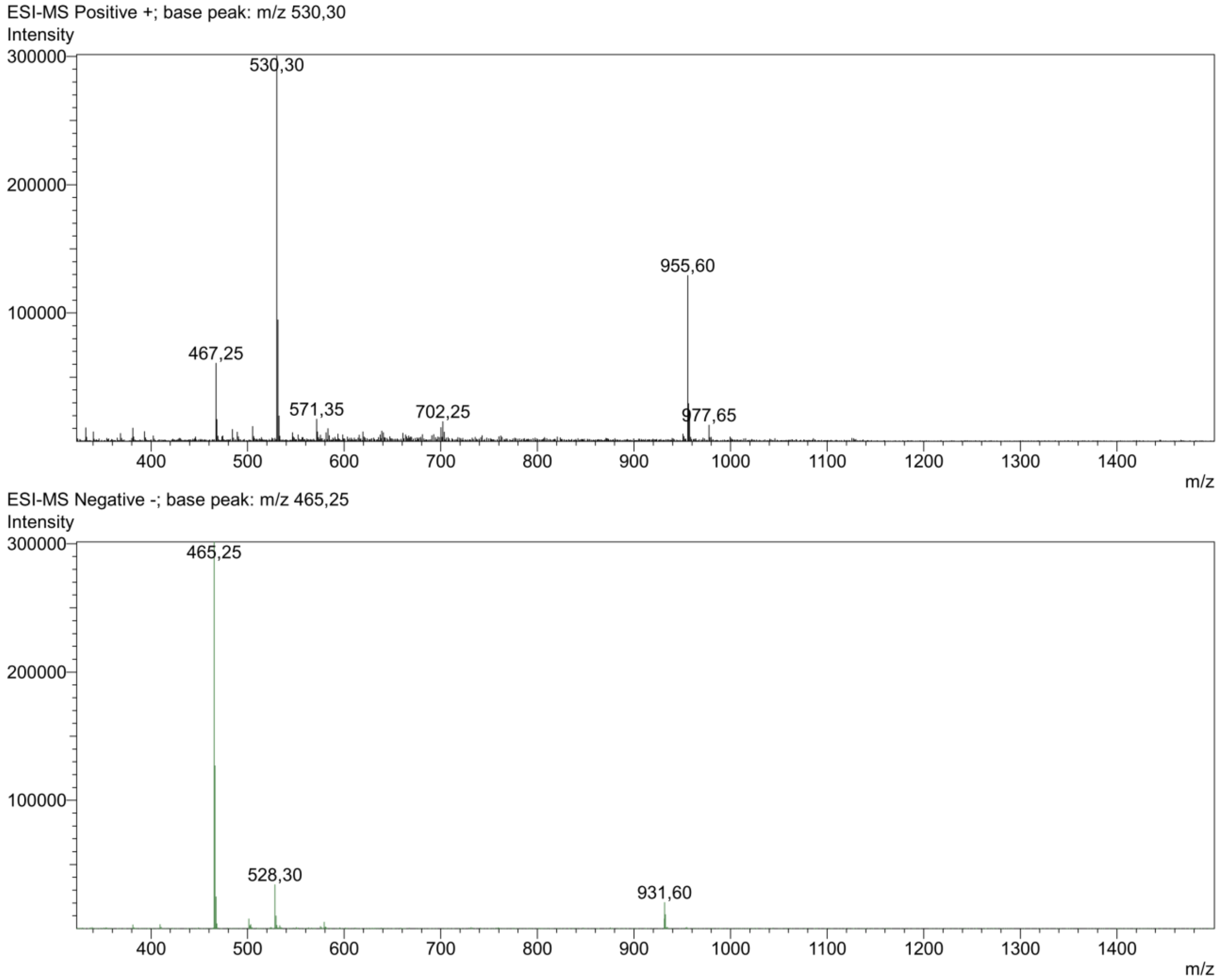

Figure S4. ESI-MS spectra of *n*-octyl-chlorogenate, mw 466.22 g/mol.

n-dodecyl chlorogenate

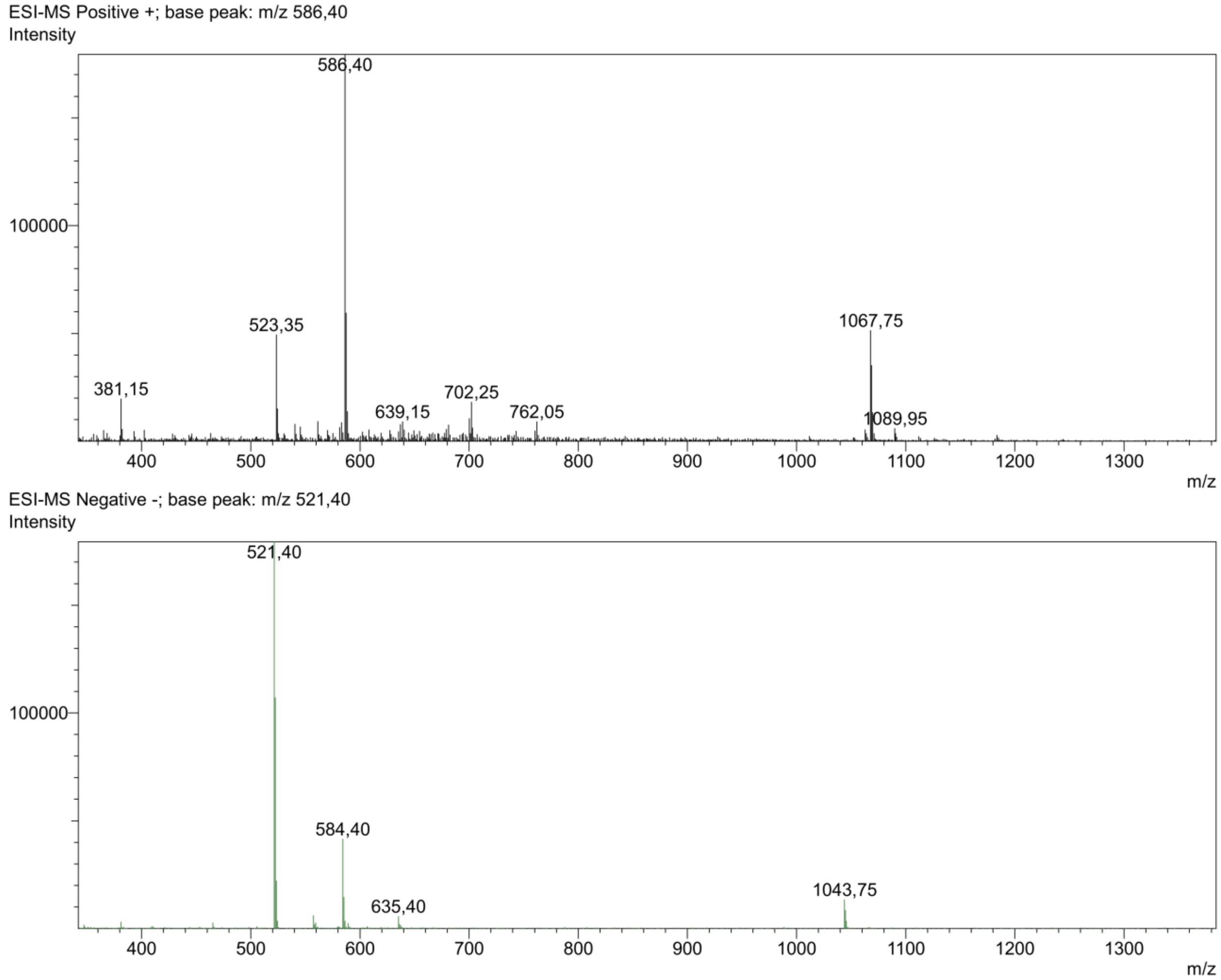

Figure S5. ESI-MS spectra of *n*-dodecyl-chlorogenate, mw 522.64 g/mol.
